# Bioplastics for Food Packaging: Environmental Impact, Trends and Regulatory Aspects

**DOI:** 10.3390/foods11193087

**Published:** 2022-10-05

**Authors:** Rui M. S. Cruz, Victoria Krauter, Simon Krauter, Sofia Agriopoulou, Ramona Weinrich, Carsten Herbes, Philip B. V. Scholten, Ilke Uysal-Unalan, Ece Sogut, Samir Kopacic, Johanna Lahti, Ramune Rutkaite, Theodoros Varzakas

**Affiliations:** 1 Department of Food Engineering, Institute of Engineering, Campus da Penha, Universidade do Algarve, 8005-139 Faro, Portugal; 2 MED-Mediterranean Institute for Agriculture, Environment and Development and CHANGE-Global Change and Sustainability Institute, Faculty of Sciences and Technology, Campus de Gambelas, Universidade do Algarve, 8005-139 Faro, Portugal; 3 Packaging and Resource Management, Department Applied Life Sciences, FH Campus Wien, University of Applied Sciences, 1100 Vienna, Austria; 4 Department of Food Science and Technology, University of Peloponnese, 24100 Kalamata, Greece; 5 Department of Consumer Behaviour in the Bioeconomy, University of Hohenheim, Wollgrasweg 49, 70599 Stuttgart, Germany; 6 Institute for International Research on Sustainable Management and Renewable Energy, Nuertingen Geislingen University, Neckarsteige 6-10, 72622 Nuertingen, Germany; 7 Bloom Biorenewables, Route de l’Ancienne Papeterie 106, 1723 Marly, Switzerland; 8 Department of Food Science, Aarhus University, Agro Food Park 48, 8200 Aarhus, Denmark; 9 CiFOOD—Center for Innovative Food Research, Aarhus University, Agro Food Park 48, 8200 Aarhus, Denmark; 10 Department of Food Engineering, Suleyman Demirel University, 32200 Isparta, Turkey; 11 Institute for Bioproducts and Paper Technology, Graz University of Technology, Inffeldgasse 23, 8010 Graz, Austria; 12 Sustainable Products and Materials, VTT Technical Research Centre of Finland, Visiokatu 4, 33720 Tampere, Finland; 13 Department of Polymer Chemistry and Technology, Kaunas University of Technology, Radvilenu Rd 19, 50254 Kaunas, Lithuania

**Keywords:** food packaging, bioplastics, environmentally-friendly, consumer perception, biodegradation, sustainability

## Abstract

The demand to develop and produce eco-friendly alternatives for food packaging is increasing. The huge negative impact that the disposal of so-called “single-use plastics” has on the environment is propelling the market to search for new solutions, and requires initiatives to drive faster responses from the scientific community, the industry, and governmental bodies for the adoption and implementation of new materials. Bioplastics are an alternative group of materials that are partly or entirely produced from renewable sources. Some bioplastics are biodegradable or even compostable under the right conditions. This review presents the different properties of these materials, mechanisms of biodegradation, and their environmental impact, but also presents a holistic overview of the most important bioplastics available in the market and their potential application for food packaging, consumer perception of the bioplastics, regulatory aspects, and future challenges.

## 1. Introduction

Packaging is an integral part and enabler of modern food systems. As a result, there is hardly any food item today that is not packaged at least once on its way from farm to fork [1,2]. The background to this is the underlying and essential service functions that it performs. Even the most trivial function, namely containment, is what makes liquid foodstuffs, for example, manageable and transportable in the first place—a key function for our modern economy. Moreover, and most importantly, it provides protection to the food, thus, enabling high levels of food quality, safety, and security to be achieved. This is rounded off by the functions of communication (e.g., information about the product) and convenience (e.g., easy-to-open) [3].

The needs of a food product are strongly dependent on the type of packaging (e.g., design, type of construction) and packaging material chosen (e.g., paper, glass, metal, and corrugated or non-corrugated cardboard, plastic, and composite materials with more than one material, such as plastic-coated cardboard). Hence, careful consideration of the material’s properties is a key step in designing packaging that is fit for its purpose and, thus, effective. Properties include features, such as a barrier against gasses (e.g., oxygen, carbon dioxide, water vapor), physical and mechanical strength, aroma, fat, lightness, and migration, as well as hygiene and, as a result, are strongly dependent on the nature of the material itself [3,4,5].

Taking a closer look at plastic materials, it quickly becomes clear this material group comprises a wide range of different materials, including polyolefins, such as polyethylene (PE), polypropylene (PP), and polyethylene terephthalate (PET), each with very different properties. Accordingly, they also offer a wide range of advantages and disadvantages. In terms of suitability for packaging applications, it can be said that plastics are often preferred because of their lightness, formability, low cost, versatile and controllable properties (e.g., mechanical, physical, and chemical properties, barrier, color, temperature stability, and sealability), convenience (e.g., transportability and resistance to breakage) and usability in the preparation of multilayer materials [3,4,5]. Despite the, per se, very good suitability, it is above all the environmental aspect and the careless handling of raw materials and packaging waste, such as (marine) litter, microplastics, limited recyclability and (bio)degradability, and the use of fossil resources, that pose a major disadvantage and have been the focus of public and political debate in recent decades [6,7,8,9].

Among different sectors, the packaging sector is the main user of plastics (around 40%). For example, plastic packaging in the European Union (EU) makes up around 60% of post-consumer plastic waste [10]. Most of the packaging is used only once, and the lack of reuse associated with failures in the recycling systems contribute to generating huge amounts of solid wastes that are discarded, contributing to a negative impact on land and marine environments [11]. On average, the amount of plastic packaging waste generated per capita increased from 27 kg to 35 kg between 2009 and 2019 [12].

The EU is trying to solve these problems with approaches, such as circular economy and bioeconomy, to promote innovation and research for guaranteeing resource utilization efficiency. The circular economy highlights the 4R concept (reduce, reuse, recycle, and recover), and stresses that sustainable production and consumption of resources should be developed and used where the evidence clearly shows that they are more sustainable compared to conventional petrochemical plastic production. The bio-economy is related to the renewable part of managing agricultural waste [8,13]. Furthermore, the United Nations 2030 Agenda for Sustainable Development aims, among other goals, to substantially reduce waste generation through prevention, reduction, recycling, and reuse (Goal 12.5) and to prevent and significantly reduce marine pollution of all kinds, in particular from land-based activities, including marine debris and nutrient pollution (Goal 14.1) [14].

Thus, one of the challenges to our society is to decrease the amount of durable and non-biodegradable packaging materials, such as glass, metal, and mainly plastic, and to find new solutions. The search for viable alternatives with suitable packaging properties is continuously under study, and the reduction in these wastes can be achieved with the development of new environmentally friendly packaging systems [11].

New packaging systems with bioplastics have been developed in the last two decades. This packaging includes materials derived from renewable resources and/or biodegradable polymers, and ranges from flexible films to rigid materials that have a high potential to produce sustainable packaging. These bio-materials are usually blended to control and achieve desirable mechanical, physical, and barrier properties [15]. Although cultural, economic, and even culinary factors from different geographic areas may contribute in different manners to shaping and selecting these different environmental friendly materials [16], the main objectives of this work are to present a literature review of the different properties of these materials, regulations, and mechanisms of biodegradation, to create a holistic overview of the most important bioplastics available in the market at an international level and their potential application for food packaging, environmental impact, a systematic review how consumers perceive bioplastics, and future trends.

## 2. Definitions and Regulations 

**Figure 1:**
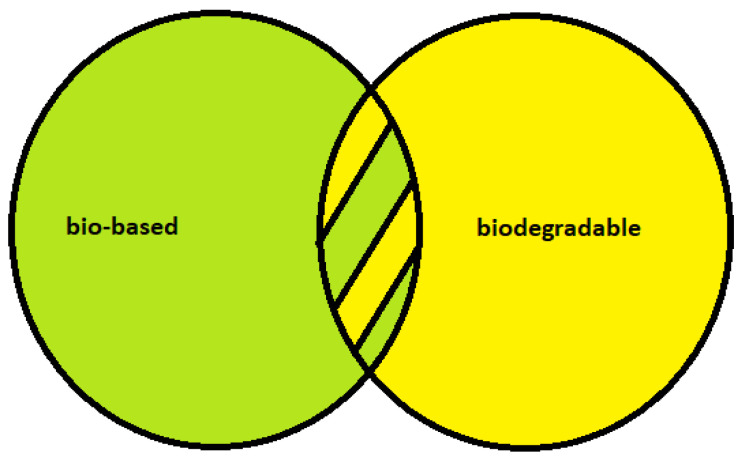
Bioplastics are bio-based, biodegradable, or both (adapted from European Bioplastics [17]).

According to the European Bioplastics Organization (EBO), the term ‘bioplastics’ refers to both the bio-based origin of plastic and/or its biodegradable character (Figure 1) [17]. Those derived from plant-based materials (also known as biomass) are bio-based plastics according to the European Standard EN 16575 from 2014 [18,19]. However, it is not only bio-based plastics that are biodegradable, and not all types of bio-based plastics are biodegradable [18], as will be discussed in Section 3.

Hence, we should appropriately define the vocabulary surrounding bio-plastics. From a chemical point of view, and in contrast to the most frequently used types of plastics worldwide (polyolefins are by far the most abundant [20]), the vast majority of substances among biopolymers are linked via heteroatom bonds. This is due to the fact that selective linkage of C–C bonds is chemically very challenging, and regioselective cleavage of non-polarized bonds even more so [21,22]. In nature, reversibility and energetically favorable activatability are essential in the enzymatically catalyzed biosynthesis of structural and storage polymers (in fact the utilization of artificial enzymes for chemical synthesis is an increasingly studied field, with the potential to shift synthetic chemistry toward more environmentally friendly and less energy-intense methods) [23]. This is usually based on nucleophilic substitution of carbon centers (mostly carbonyl or acetals/ketals) positively polarized by doubly- or singly-bound oxygen, with the linkage of C–O or C–N hetero bonds. In addition, the monomer building blocks must be capable of aqueous solvation to enable polymerization and are activated with suitable leaving groups to provide the energy needed for biosynthesis (typically nucleotide activation of building blocks, such as carbohydrates or amino acids). The substitution reactions are catalyzed by selective enzymes, such as peptidyl transferases [24], glycosyl transferases [25], or polyester synthases [26], while enzymatic polymerization, as well as artificial enzymes, are also important objects of research [27,28]. Less polar monomers, such as lignin precursor molecules, are typically conjugated with polar compounds, such as carbohydrates, to enable transport in the cytosol, which is mandatory for the further biosynthesis of wood [29]. This results in the classes of substances available as biopolymers, most of which are derived from functionalized carbonyl groups. These include carboxylic acid derivatives, such as proteins or polyesters, and acetals/ketals, such as carbohydrates. Due to the aforementioned requirements for monomers and enzymatic reactions, namely water solubility and the possibility of forming hetero-bonds, an increased functionalization with polar groups, such as alcohols, amines, or carboxylic acids, is found and, thus, a tendency towards the polar character is identified. This results in significant physicochemical properties of the material due to the increased intra- and intermolecular interactions, which influence processability, barrier properties, and several other factors. These properties include higher crystallinity and melting or glass transition temperatures, whereby the intermolecular interactions outweigh the intramolecular interactions due to strong hydrogen bonds (strong interactions lead to higher heat resistance but also a higher tendency of water absorption) in the extreme case of carbohydrates. This results in decomposition instead of melting, and the number of hydrogen bonds must, therefore, be reduced either by additives, e.g., when obtaining thermoplastic starch (TPS), or by chemical modifications to enable thermoplastic processability [30]. An important exception to this is lignin, which contains a mixture of phenolic ethers and radically linked carbons, i.e., it is comparable to phenolic resins, such as Bakelite, can be used as a basis for similar materials, and, thus, has a much more apolar character, as well as poor water solubility [31,32,33]. The typical thermoset networks are, therefore, particularly stable and also require organisms capable of degrading lignin to expend more energy than other biopolymers. This, and the inhomogeneity of the material, also depending on the starting material, have led to the fact that lignin has hardly been used for packaging so far, despite its abundance and inexpensive availability. Nevertheless, it has a lot of potentials to be utilized for water vapor barrier functionality [34].

In relation to the bio-based origin, there is no general agreement on a specific reference limit; however, threshold values of renewable content that mark the bio-based nature of a material can be found in national regulations [35]. For example, the United States Department of Agriculture (USDA) BioPreferred Program depended on product category factors to determine a wide range of minimum acceptable bio-based content of between 7–95% [36]. However, certifiers, such as the certification organization of TÜV Rheinland, the German Technical Inspection Association, and DIN, the German Institute for Standardization (DIN CERTCO), and the Technical Inspection Association (TÜV) AUSTRIA Belgium, provide standardized labels that indicate the biomass content of bio-based materials [35,37].

According to the European Commission (EC) policy recommendation, waste-to-energy (WtE) processes respect the waste hierarchy, making co-combustion processes energy-efficient techniques. This leads to the maximization of the circular economy’s contribution to decarbonization [38].

The EU has addressed the problem of plastic food packaging in its plastic strategy and Circular Economy Action Plan [39]. The transition towards a circular economy is offered as a comprehensive solution for the plastic crisis. This requires various collaborations and the engagement of different societal actors, such as citizens and consumers, authorities, policymakers, and non-governmental organizations (NGOs), whose aim is the creation of novel producing methodologies for packaging materials and the manufacturing of sustainable foods.

The negative environmental impacts have raised increasing concerns, both in public media forums and in the cabinets of policymakers [8]. Several policies and regulation measures include the reduction or ban of single-use plastics [40]. Voluntary measures, such as collaborative commitments [41] and pacts [42] to foster the circular economy of plastics, have been proposed by public and private bodies to address the problems caused by plastic food packaging. 

Since the establishment of the United Nations’ 17 sustainable development goals (SDGs) [43], many companies have advocated sustainable practices. These goals aim to make use of renewable sources without causing impacts on human health (SDG3), climate change (SDG13), to preserve life below water in oceans, seas, and marine resources for sustainable development (SDG14), and to protect life on land (SDG15). Circularity is one of these goals which aims to tackle SDG11 (sustainable cities and communities) and SDG12 (responsible production and consumption). However, the transition toward environmentally-friendly plastics following the adoption of the SDGs is still slow and requires country-specific policies.

This is due to the many choices and approaches followed by producers, consumers, and policy-makers. A shift towards the circularity and sustainability of plastics is required.

Policy measures are essential for the management of plastic waste and mitigation of its generation. They should be enforced at all stages of collection, storage, transportation, and final disposal or recycling. Of course, these policies should be financially sustainable, and technically sustainable, and should incorporate social, legal, and environmental aspects [44].

These measures will include prevention strategies for the reduction in waste and control of types of waste and materials through bans, restrictions, and control strategies by the adoption of standards and protocols, and practices on the ground. Allocation of different roles and responsibilities for each party among stakeholders is also essential [45,46,47].

The Chinese waste import ban of 2017 showed the highest impact on the reduction in plastic waste. This pushed several countries to find other solutions for their plastic waste.

Table 1 shows the percentage of imports and exports of plastic waste referring to some European and non-European countries, while Table 2 shows the countries with regulations about types of banned plastic materials.

The EU-28 represents the largest exporter of plastic waste, accounting for around one-third of all exports of plastic waste from 1988 to 2016 [50]. Most of this waste has now been halved and re-routed to Vietnam, Thailand, and Malaysia [51].

**Table 2:** Countries with regulations about types of banned plastic materials.

Countries	Level	Types of Banned Plastic Materials	References
Canada, Costa Rica, Taiwan, Belize, India, and the USA (California and Florida)	National bans	Single-use plastics (SUPs), including plastic bags, straws, and cutlery	[52]
The Netherlands, Tanzania, Australia, Italy, South Korea, New Zealand, the UK, the USA, and Canada	National bans	Microbead plastics	
25 African countries	National bans	Plastic bags	[53]
Australia	National bans	Lightweight plastic bags	
Papua New Guinea	National bans	Nonbiodegradable plastic bags	

Retailers have taken voluntary actions to reduce plastic bag consumption within the European Union. For instance, many supermarkets have voluntarily abolished the provision of (free) plastic bags (such as in Austria and Lithuania) and others have introduced a value of around EUR 0.05–0.10 per single-use plastic bag (Belgium, Estonia, France, Germany, Hungary, Latvia, the Netherlands, Portugal, Sweden, Slovakia, and the UK) or have substituted them with biodegradable plastic bags (Austria, France, and Sweden) or used alternative bags made of cotton, hessian, or linen. Plastic pollution of the environment can be reduced by interventions, such as ‘Operation Clean Sweep’, organized by non-governmental organizations (NGOs) to clean beaches and drains [54]. Reusable bags are produced by NGOs who sell them to finance their activities in part. Raising awareness through media campaigns or billboards to remind customers to reuse their bags is another strategy adopted by the UK. Finally, paying customers a small amount of money (around EUR 0.10) if they do not take any plastic bags is supported in the UK [44].

Extended producer responsibility (EPR) is another policy mechanism that aims to mitigate the risks associated with waste management. With EPR, the mitigation of the environmental impacts of products throughout their lifecycle stages is accomplished by producers who are legally and financially responsible. Indeed, EPR can help in plastic pollution prevention and mitigation by limiting the health, safety, environmental, and social impacts of plastic products [55]. However, difficulties with enforcement have been reported.

Hence, the implementation of recycling processes and the development of biodegradable plastics are some of these strategies. Europe halved its monthly plastic waste export with these restrictions (from 300 to 150 kton) [49] and, in 2019, the Basel Convention called for more domestic solutions in dealing with (hazardous) waste [56]. This is signed by 187 countries worldwide (excluding the US, among others). 

At the European level, the new EU Green Deal 2020 is targeting (illegal) waste exports to third countries. At the same time, a regulatory framework for biodegradable and bio-based plastics is set to be implemented aiming at the local improvement of waste management techniques and leading to the push of recycling processes forward, hence, reducing the need for biodegradable plastics. The development of both circular and bio-economies will be implemented by the amelioration of rural areas with a new financial plan [57].

Financially speaking, setting clear criteria for the assessment of green investment funds is one of the goals of the 2018 EU regulation facilitating sustainable investment in this direction [58,59]. Europe imposes fees to discourage plastic production under the extended producer responsibility (EPR) concept [60]. Moreover, the European Chemical Agency (ECHA) has recently discussed intentionally-added microplastics (e.g., microbeads in cosmetics) by the provision of a socio-economic assessment [61].

Substances of very high concern (SVHCs, i.e., carcinogenic, mutagenic, or toxic for reproduction, or CMR, and persistent and/or bio-accumulative substances) are being banned by the REACH regulation at the EU level [62,63] due to the cumulative and detrimental effects of (micro) plastics. In the future, the EU Green Deal [6], as well as the REACH registration of polymers, might aid in the classification and management of hazardous substances in (new) waste streams.

Currently, California law wants to phase out plastics that cannot be compostable or recyclable, but even this legislation faces bureaucratic resistance [64]. Other countries, such as China, support research on biodegradable plastics via funding, but also have limited policies [65].

California developed policy concepts in 2013 to make the producers of selected products responsible not only for recycling but also for litter prevention and mitigation. This new policy required a reduction in their products’ total volume in the environment by 95% in 11 years [55]. Bureaucracy might be a major obstacle in achieving these goals. Moreover, it might work well for some products but not for others. Difficulties with enforcement might also occur, and the problem of data scarcity has been reported in developing countries [66].

Finally, political will might be lacking due to countries having other priorities. Some ways to promote the political will are to make this the priority of the country analyzing the impact of environmental changes on health and society. Governments should employ tools that allow all consumers to enhance their awareness of the management of plastic and plastic waste. Consumers should change habits and lifestyles that require plastic usage, e.g., by means of a reduction in the reliance on single-use plastics or through source preparation and social awareness, and public education programs should also be included [44].

## 3. The Common Misconception in the Definition of Biodegradable and Compostable Polymers

Degradable polymers are polymers that disintegrate by different mechanisms, including physical, chemical, and/or biological processes, resulting in a loss of some properties that may vary as measured by standard test methods appropriate to the plastic. A biodegradable polymer is defined as a polymer that undergoes degradation due to the action of various microorganisms within a specific period and environment. A compostable polymer is a polymer that is degraded by biological actions during composting to yield carbon dioxide (CO_2_), water (H_2_O), and inorganic compounds. However, the terms “biodegradable” and “compostable” may lead to confusion among consumers and other stakeholders. The simplified distinction between the two terms is accepted that all compostable plastics are biodegradable but not all biodegradable plastics are compostable, so the two terms are not to be used interchangeably. In addition to these two main terms, there are some other complex definitions, such as home compostable, industrial compostable, and marine-degradable, regarding biodegradable polymers. *Industrial compostable polymers* are composted under a controlled process (very strictly controlled oxygen, water, and heat input) in industrial composting plants to be used in agricultural applications, while *home compostable polymers* are defined as polymers that can fully decompose in the soil [67]. On the other hand, *marine-degradable* plastics are plastics that can be degraded into CO_2_ and H_2_O_2_ in marine environments, including coastal and ocean waters, lakes, lake-connecting waters, subsoils, submerged lands, and sea and coastal habitats, under light, heat, or microbial effect. However, a harmonized EN standard for only industrially compostable packaging exists, whereas no general standard for marine biodegradation is implemented. Currently, no detailed EU law is present for bio-based, biodegradable, and compostable plastics. The EU Commission announced a policy framework where resources of bio-based feedstock and the environmental benefits of using biodegradable and compostable plastics will be evaluated, as well as the conditions for these uses [68].

### Brief Overview of Degradation Pathways for Polymers

At present, the complexity of biodegradation is accepted, as it includes several steps, such as biodeterioration, depolymerization, assimilation, and mineralization [69]. The biodegradation steps and mechanisms behind this process have been exclusively addressed elsewhere [69,70,71,72,73]. In this part, a very brief overview of degradation pathways is provided, which is then to be associated with the environmental impact of bioplastics.

**Figure 2:**
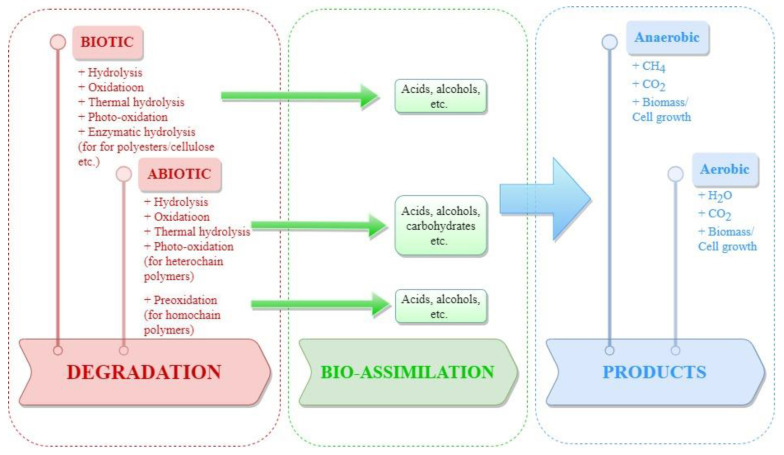
Theoretical biodegradation pathway for polymers (adapted from Meereboer et al. [70]).

Biodegradation is a process that degrades materials into CO_2_, H_2_O, biomass, and CH_4_ with the help of living microorganisms under various environmental conditions, such as compost, soil, marine conditions, or other mediums [74]. Abiotic degradation, such as oxidative or hydrolytic degradation, may initiate or enhance biodegradation by increasing the surface area of the organism–polymer interface [69,75,76]. In general, enzyme-catalyzed or biotic reactions are efficient methods for the biodegradation of polymers. Furthermore, after the abiotic and/or biotic degradation of polymers, the final products are bio-assimilated by microorganisms to be used as growth factors or in cellular respiration (Figure 2) [70].

Polymer biodegradation results in various products depending on whether it occurs under aerobic or anaerobic conditions. As mentioned earlier, in aerobic degradation, oxygen is utilized as the final electron acceptor while, in anaerobic degradation, CO_2_, nitrates, or sulfates are used as the electron acceptors by microorganisms to produce the energy needed to maintain cell functions [77,78]. However, most of the biodegradable polymers biodegrade under both aerobic and anaerobic conditions [79] and, in enzymatically degradable polymers, such as PLA (polylactic acid), temperature plays an important role in how polymer scission occurs [71].

Aerobic biodegradation is the conversion of organic carbon into CO_2_ and water as a result of microbial metabolism in the presence of oxygen. In anaerobic biodegradation, methane is produced, while some CO_2_ can be obtained depending on the residual oxygen or the type of degraded material. Soil biodegradation, composting, and marine biodegradation are the main areas of aerobic biodegradation standards, whereas sewage sludge biodegradation, anaerobic digestion biodegradation, and (accelerated) landfill biodegradation are the main areas of anaerobic biodegradation standards [70]. Landfills may result in the uncontrolled biodegradation of plastic materials with methane release to the environment, while biogas facilities are a part of anaerobic digestion systems, capturing the released methane for energy conversion [80]. Inappropriate applications in the biodegradation of polymers may result in methane release in the environment due to the switching from anaerobic to aerobic conditions [70].

## 4. Research on Bioplastics 

The lifespan of plastics produced from petrochemicals has been proven to be several decades, and the need to replace them with bioplastics is more urgent than ever. For example, packaging materials made of PET (such as beverage bottles) have a proven lifespan of more than 90 years [81].

The production of biopolymers is based on living organisms and takes advantage of various properties, such as strength, stability, and flexibility. Plants, crops, animals, and microorganisms are the basic raw materials that can be used to produce biopolymers [82]. Producing innovative bioplastics using biological raw materials is expected to lead to significant benefits in certain areas, such as the environment and the economy [83]. The classification of biopolymers into different categories can be carried out in different ways, since the number of resources from which they arise is extremely large [84]. A classification system concerns the division into categories based on how biodegradable they are and according to their biomass content. Based on these criteria, there are (i) bio-based and non-biodegradable, (ii) biodegradable and bio-based, and (iii) biodegradable and fossil-based alternatives [85]. Another classification can be made according to the origin of the resources, which means that it is possible to have biopolymers derived exclusively from renewable resources and polymers which are mixtures of biopolymers and commercial polyesters [84]. Bio-based and biodegradable biopolymers can also be categorized into synthetic biopolymers (synthesized from bio-derived monomers), microbial biopolymers (produced by microorganisms), and natural biopolymers (extracted from biomass) [86]. Polysaccharide-based films, protein-based films, or a combination of both are the biopolymers with the greatest potential in film making. In food packaging, important pathogens, such as *Listeria monocytogenes*, *Salmonella*, *Campylobacter*, *Bacillus cereus*, *Saccharomyces cerevisiae*, *Staphylococcus aureus*, *Aspergillus niger*, and *Clostridium perfringens*, may survive and develop depending both on the conditions inside the packaging but also on the conditions of the external environment of the packaging. Much biodegradable green packaging has significant antimicrobial functions due to the bioactive compounds contained in plant by-products [87]. 

### 4.1. Protein-Based Bioplastics 

Protein-based bioplastics can be derived from raw materials of both plant and animal origin. Common sources of plant origin are wheat gluten, soy, pea, corn zein, and cottonseed proteins. On the other hand, whey, casein, collagen, gelatin, and keratin are some proteins of animal origin [88]. Because proteins consist of different types of amino acids, the strong intermolecular binding of proteins affects the functional properties of protein-based bioplastics, giving them superior characteristics in comparison with carbohydrates and lipids [89]. Protein-based films are extremely popular, as they are abundant, inexpensive, non-ecotoxic, biodegradable, and have very good film-forming properties [90].

### 4.2. Polysaccharide-Based Bioplastics

Polysaccharides have also been proposed as a biopolymer source for bioplastics [91]. Alginate, cellulose, pectin, and starch are derived from plants, while glycogen and chitin are of animal origin [92]. 

#### 4.2.1. Cellulose-Based Bioplastics

Cellulose is the most abundant biopolymer available on the planet, gaining an important role in the production of new materials. Cellulose is renewable, widely available, non-toxic, low-cost, environmentally friendly, biocompatible, biodegradable, thermally and chemically stable, and derivable [93,94]. Fruit and vegetable waste is very rich in this valuable biopolymer. Cellulose esters and cellulose ethers are the main cellulose derivatives that are used in industrial applications, as the production of pure cellulose bioplastics still remains quite difficult, due to the structural complexity and difficulty in melting and dissolving it through standard processes [95]. Mechanical properties, thermal stability, and water absorption are some properties of bioplastics that could be improved with the addition of cellulose [96].

#### 4.2.2. Starch-Based Bioplastics

Potato is the main source of starch for the production of bioplastics. Cereals and legumes, such as wheat, rice, barley, oat, corn, beans, and soy, are also significant sources [97]. Starch must be incorporated with many plasticizers, as the main problem with starch in the food packaging industry is its low plasticity [98].

### 4.3. Synthetic Bioplastics

The main synthetic bioplastics are PBS (polybutylene succinate), PLA (polylactic acid), PVOH (polyvinyl alcohol), PGA (polyglycolic acid), and PCL (polycaprolactone), [97].

Indeed, PLA is one of the most commonly used bioplastics and, in the year 2021, had the largest market share for the production capacity of biodegradable bioplastics worldwide [99].

On the other hand, PCL is easily processable, belongs to semi-crystalline polymers, and is fully biodegradable. As a result, 11% of the total market of biodegradable polyesters is held by PCL. It is a bioplastic with excellent compatibility with other polymers and additives, which makes it very promising in food packaging in the future. The PGA bioplastic has a similar chemical structure to PLA, but it is characterized by improved degradability, mechanical properties, and gas barrier properties that make this a beneficial supplement to PLA. Indeed, PBS is extremely flexible, elastic, and biodegradable, with a low glass transition temperature. Another bioplastic, PVOH, is widely used for food packaging due to its good film-forming ability, biodegradability, non-toxicity, water processability, and low cost [100].

The following tables (Table 3 and Table 4) present studies on bioplastic materials for food packaging and their properties developed using fruit and vegetable by-products during 2017–2021. European countries, the USA, China, and India are among the countries that contributed to the development of these bioplastic materials.

The presented research studies from the last five years show the great potential of these types of materials. The following section will present the existing main types of utilizations for bioplastics in packaging materials, their main properties, and their applications at an industrial level.

## 5. Applications 

In general, there are four main types of utilization of bioplastics in packaging materials, as follows:

1. Structural material—bioplastic is used as a mono-material, for polymer blends, or in composite materials [123,124,125];
2. Coating—bioplastic is used as a coating on the substrate material, forming a multi-layer material to increase barrier functions, enhance processability (printability and sealability), functionalize the surface, or serve another duty. Typically, a coating is accomplished by extrusion, film casting, or common lacquer application techniques [126];
3. Additive—biopolymers can be utilized as functional additives in plastics to control physico-mechanical properties, such as strength, stiffness, hardness, or barrier functions (e.g., nanocellulose diffusion barriers) [127,128];
4. Filler—bio-based materials serve as fillers that can reduce material costs and/or increase the ratio of renewable resources in bioplastic packaging materials [129].

Holistic approach for material selection

In general, there are a number of different, at least partially, plastic-based packaging systems. In this context, plastic can be represented either structurally or as a functional coating. The applicability of different plastics is primarily limited by mechanical material properties, which in turn can be derived from the molecular basis. These include, for example, rigid trays (T), bottles (B), pouches (P), coated cardboard (C), films, and wraps and bags (F) [97,128].

### 5.1. Processing

There are a number of different processes for manufacturing the various types of plastic-based packaging products mentioned above, each of which has specific requirements for different physical material properties with special emphasis on rheology [130]. Plastic melts are non-Newtonian shear-thinning (viscoelastic) fluids [131]. Due to the disentanglement and realignment of the molecules under high pressure, a drop-in viscosity and pseudo-plastic behavior are observed [132]. Typically, methods, such as melt flow index (MFI) measurement, are used to provide fast conclusions about chain length and melt viscosity (where lower values of the same polymer typically correspond to higher viscosity and higher chain length) [133].

For example, for injection molding, sufficient fluidity of the melt must be ensured to fully penetrate the mold [134,135], whereas for extrusion, due to the absence of such a mold, a higher viscosity is advantageous for stability. It is noteworthy that thermal and mechanical processing parameters, as well as throughput rate, may affect material degradation during processing [136]. Therefore, the desired type of packaging and the associated manufacturing method(s) play an essential role in material selection.

Limitations in applicability due to the molecular basis (caused by properties, such as brittleness) are addressed via variations in molecular weight or side-chain length, fillers, additives, plasticizers, blending with other types of polymers, and/or co-polymerization, resulting in different polymer grades and types of plastic tailor-made for various processing methods. It is important to point out that higher amounts of additional components may affect the recyclability of the material and that additives should be chosen carefully to minimize environmental harm after being littered [137].

In the following, the different processing approaches are described:

Extrusion coating and film production (casting and blown film)

In the extrusion process, previously compounded materials are fed into a screw barrel equipped with a screw conveyor, melted, compacted, and pressed via a die through a 2D shaping profile die to produce a continuous polymer strand whose cross-section corresponds to the applied die and which can optionally also contain cavities [138]. For packaging, cast film and tube extrusion are particularly relevant. In extrusion, the flow behavior is decisive for the quality of the product. The use of longer-chain and, therefore, higher-viscosity grades tends to reduce the risk of deformation in the obtained extrudate [139]. In addition, the molecular structure is decisive for the crystallization behavior and, thus, besides processing parameters, influences the sharpness of the melting range. Having control over crystallization behavior is an important aspect of polymer engineering [140]. Cast films typically have lower crystallinity due to rapid cooling and, thus, usually have better transparency and gloss [141]. The method is well-suited for thicker films that are subsequently further processed via thermoforming [142].

Injection molding (I)

Similar to extrusion, pre-compounded plastic is melted and compacted by a screw and conveyed to the injection nozzle. Instead of a profile mold, the material is pressed into an injection mold, allowing 3D structures to be made from plastic. For the process, with higher complexity of the injection mold, good flowability of the material is essential so that the mold is completely and uniformly filled. Furthermore, process parameters, such as mold temperature, significantly affect mechanical properties [143].

Thermoforming (T)

Here, 2D plastic films (semi-finished products) are continuously processed into a stable 3D shape by thermal softening in the elastic range above the glass transition temperature and with the aid of a cooling tool, whereby the process is usually supported by vacuum or compressed air. The films or sheets are clamped to ensure forming with wall thickness reduction [142]. After filling with a sealing film, thermoformed cups and trays are usually sealed by using pressure and spot heating above the melting temperature, whereby chemical compatibility and a similar melting range must be ensured for the material’s combination as a basis for homogeneous bonding [144].

Blow molding (B)

In blow molding processes, preforms produced by injection molding are blown into a mold (e.g., PET bottle production) [145] or tubes are extruded and blown into films using ring dies coupled inline to an extruder (e.g., PE bag production) [146].

### 5.2. Properties

A huge variety of material properties need to be analyzed before a rational decision for a certain material can be made. This decision depends on specific barrier requirements of the packed food and other factors, such as ecological and economic criteria. Often, despite a favorable low price, no clear general pro or con can be formulated for different packaging types. Since mechanical properties affect the processability, materials that are applicable for injection molding may be unsuitable for extrusion and vice versa. Physical and mechanical properties are interconnected and a result of underlying chemical structures of the biopolymers, additives, and fillers, and their inter-and intramolecular interactions. Crystallinity correlates directly with properties, such as brittleness, tensile strength, and gas and aroma permeability. Furthermore, permeability is dependent on solubility that is a function of polarity [147].

#### 5.2.1. Biodegradability

As previously presented in Section 3, the term biodegradable implies that microorganisms can completely degrade a material into elementary components or small molecules within a specific period and environment. Depending on environmental conditions (such as pH, temperature, and/or oxygen availability) resulting in differences in the microbial colonization of diverse habitats, different categories can be used to describe biodegradability. Additionally, standardized test methods are available and can be used to characterize it. However, some of these methods, such as solely analyzing weight loss over time, do not give a sufficient direct proof of biodegradation [148].

Four common categories are proposed that are typically used to describe the behavior of different materials in the context of biodegradability. Here, classification in a lower category automatically corresponds to “upward compatibility” for higher categories (except category 4). These categories are as follows:

Category 1—marine biodegradable (claimed to be biodegradable in the marine environment);
Category 2—home compostable (claimed to be biodegradable in soil without optimized composting conditions);
Category 3—industrially compostable (according to EN 13432);
Category 4—non-biodegradable (within the time frame specified by definition).

It is important to note that especially Categories 1 and 2 currently cannot be sufficiently backed up with standardized methods that allow reliable forecasts for estimating the degradation time in the natural environment. Determining the transferability of defined laboratory conditions is, in many cases, not possible or possible only to a limited extent due to the complexity and abundance of influencing parameters, as stated by Choe et al. [149] in their review which compared results from laboratory and environmental experiments.

Currently, little is known about the ecotoxicological impact of biodegradable micro- and nano-plastics. Increased degradation rates increase the amount of micro-bioplastics coming from biodegradable polymers that pose certain risks, such as shifts in microbial communities (that could destabilize delicate ecological balances). Microplastics from degradable polyesters, such as PLA and PHB (poly-3-hydroxybutyrate), were found to have negative effects on marine benthic communities [150]. A comprehensive recent review by Fan et al. shows that biodegradable microplastics can show more severe effects compared to conventional microplastics [151]. The release of micro- and nano-plastics into the environment during biodegradation will be discussed in more detail in Section 6.2.

#### 5.2.2. Barrier Functions

Barrier functions against gases play a very important role in the selection of materials for food packaging. If a packaging material does not offer an adequate barrier, this can lead to untimely spoilage of the contents (for example, oxidation of sensitive fatty foods caused by an inadequate oxygen barrier [152] or premature wilting of lettuce due to an inadequate water vapor barrier [153]). As already mentioned in Section 4, the permeability is determined by the molecular basis of the material. In this context, permeability is dependent on sorption and diffusion, and there is an important entanglement between sorption and the polarity of a material. Moreover, crystallinity, for example, plays a role in the diffusion process within the phase. A wide palette of measuring methods is available as reviewed in detail by Baschetti et al. [154]. The permeability of a material is a key limitation in the substitution of typical petro-based plastics, such as polyolefins [155] and, where appropriate, it is shifted by combining the plastic with orthogonally effective materials (in the form of multilayer structures, compounds, or additives). In this case, an improvement in barrier properties comes at the potential price of reduced recyclability and/or degradability and is, therefore, a tightrope walk that should be made, taking into account additional considerations, such as life cycle assessment or local recycling infrastructure [156]. It should be noted that, in some cases, biopolymers may also have superior barrier properties, such as oxygen transmission rate (OTR), and that current packaging solutions may have higher barriers than necessary for certain products to secure their typical storage time and shelf life. To avoid potential over-packaging and to save resources in this area, re-evaluations based on storage trials are, therefore, useful in addition to material decisions based on the literature. A detailed permeability comparison between the most common bioplastics and conventional plastics was recently published by Wu et al. [157]. In addition to OTR and water vapor transmission rate (WVTR), other gas transmission rates, such as carbon dioxide transmission rate (CO_2_TR), are also relevant for certain products, but these were not addressed in detail in the review. We propose a categorization on a scale basis (powers of ten) for our overview of existing materials, as follows:

OTR—A (<1), B (1–10), C (10–100), D (100–1000), E (>1000) [cm^3^/m^2^d]

WVTR—A (<1), B (1–10), C (10–100), D (100–1000), E (>1000) [g/m²/d] at 25 °C

#### 5.2.3. Feedstock

In the context of bioplastics, the question of the underlying resources is crucial, especially in terms of sustainability. Inherently, bioplastics are obtained from renewable raw materials and are the focus of research as an approach to the transition to a circular economic model. In this context, a comprehensive accompanying life cycle assessment [158] is essential to act as sustainably as possible in the choice of materials. The gap in knowledge on detailed life cycle assessment (LCA) data needed for properly assessing bioplastics has been discussed and has become the focus of research activities [159]. Tools, such as the “Product Environmental Footprints (PEF)” system developed by the EU Commission, serve to consider a large catalog of criteria, instead of one-dimensionally looking only at CO_2_ footprints to avoid distorting the picture of the actual most sustainable solution [160]. The production of bioplastics requires resources, such as land and water, and can, therefore, compete with food or fodder production and lead to environmental pollution, for example through eutrophication [161]. Directly linked to this are food security and other SDGs that need to be considered. Therefore, it seems reasonable to present different possible feedstocks for the production of bioplastics [162,163], and the following categories were defined for the overview table:

Petrol-based (P);
Natural biomass (N);
Monomers from starch/food or feed competition (first-generation) (S);
Agricultural waste/nonfood competition land use (second-generation) (W);
CO_2_ or other feedstocks decoupled from land use (third-generation) (C).

#### 5.2.4. Price

One of the greatest current obstacles to the wider use of biopolymers as a substitute for conventional materials lies in their unattractiveness in terms of price, especially in the scenario where more expensive substitute materials do not meet the necessary barrier requirements to the same extent due to molecular differences. Especially in the case of food packaging, which belongs to the fast-moving consumer goods (FMCG) sector, profit margins are often low and, thus, the scope for increased packaging costs due to more expensive materials is correspondingly limited [164]. Nevertheless, there is strong customer demand for bio-based food packaging [165]. Four categories were defined and, as the category increases, the economic applicability shifts from potential substitute material in the FMCG sector to high-priced niche applications. The classification corresponds to the state of knowledge at the time of writing, i.e., a snapshot, and there may be transitions between different categories in either direction in the future. These categories are as follows:

Category A (0.5–2 €/kg);
Category B (2.1–5 €/kg);
Category C (6–10 €/kg);
Category D (>11 €/kg).

#### 5.2.5. Production

Bioplastics account for a small but growing share of total plastics production (2019: around 1%; 2.11 million tons [166]). In addition to price aspects, the level of production capacities is also a main factor for the security of supply and, thus, affects the choice options for the materials in question, particularly for larger production volumes, since demand exceeds the current supply on the market [164]. For this reason, annual production capacities in this review are divided into the following four orders of magnitude (as with price, these are snapshots at the time the review was written):

Category A (>100 kt/a);
Category B (51–100 kt/a);
Category C (10–50 kt/a);
Category D (<10 kt/a).

#### 5.2.6. Food-Contact Material

According to Regulation No. 1935/2004 [167], food contact materials must not transfer chemicals that are hazardous to health into food products. The approval of bioplastics for direct food contact is regulated in EU Commission Regulation No.10/2011 [168]. According to the classification, materials without direct contact can, for example, be used externally in a multilayer composite, provided that an intervening functional barrier ensures that a defined migration limit is not exceeded. Some novel materials require more detailed investigation and classification. In any case, supplementary migration measurements, mostly with simulants, on packaging prototypes are also necessary. These include, on the one hand, total migration, in which the total mass of migrated substances is quantified without detailed characterization, and, on the other hand, specific migration, in which specific contaminants, such as endocrine disruptors or carcinogens are tested for.

However, toxicological knowledge is still very limited. As an example, some recent studies suggest alterations in steroid hormone metabolism caused by acetyl tributyl citrate, a common replacement for phthalate plasticizers [169,170]. On a side note, non-intentionally added substances (NIAS) that can be a result of processing conditions or chemical reactions during food storage (e.g., under acidic conditions) should be of special concern when dealing with complex bio-based and novel materials [171,172]. Moreover, potential allergenic effects are worth investigating [173]. The following cases can be identified:

Not tested (~);
Declined (o);
Approved (+).

### 5.3. Examples

Bioplastics are rarely used as mono-materials but are typically applied as blends (in many cases compatibilizers are added to improve the miscibility) or multilayers to optimize the mechanical properties as well as barrier functions. For polar compounds, such as protic polyols (carbohydrates), modifiers, such as glycerol, are added to break hydrogen bonds and allow for thermoplastic behavior. Furthermore, additives are normally used to change the physical properties of materials. Therefore, Table 5 is based on application examples that contain the previously discussed polymers as the main structural component and do not always refer to pure material.

### 5.4. Commercial Applications and Supply Chain

As outlined in the section above, a multitude of different materials has been developed through academic and industrial research. Most of the packaging include hot and cold cups, capsules, bowls, bags, overwrap and lamination films, pouches, and containers for different types of products, such as coffee and tea, beverages, salads, potato chips, bread, yogurt, fruits, vegetables, sweets, and pasta [86,97]. Specifically, starch-based materials are used as an alternative to polystyrene (PS) in disposable tableware and cutlery, coffee machine capsules, and bottles. Cellulose is used in bio-based trays wrapped with cellulose film, and cellulose-based packaging is used for bread, fruits, meat, and dried products. Additionally, PLA can be used as an alternative to low density polyethylene (LDPE), high density polyethylene (HDPE), PS, and PET in transparent, rigid containers, bags, jars, and films for yogurts, organic pretzels, potato chips, carbonated water, fresh juices, and dairy drinks. However, PEF has a better barrier function than PET and may be used in bottles, fibers, and films, while PBAT can be used in food disposable packaging and plastic films for fresh food. Moreover, as previously referenced, several producers also use other additives, such as plasticizers, to enhance the materials’ final properties, e.g., mechanical stress and moisture [123,226,227]. However, the current bioplastic market makes up less than 1% of the entire plastic packaging market, although it is continuously growing and diversifying due to demand, R&D activities, increased environmental awareness with concerns about plastics (production and consumption), and implementation of strict environmental regulations [228]. More and more companies are looking for fully, rather than partially, bio-based alternatives, yet few performant options are available.

The main examples available are PLA (NatureWorks), PEF (Avantium), bio-PE (Braskem), bio-PET (currently only ethylene glycol, but soon expanding to terephthalic acid), PHAs, cellulose acetate, starch-based plastics (Novamont); other more niche examples are based on the latest generation lignocellulosic biomass from Stora Enso, Bloom Biorenewables, Lignopure, and Lignin Industries (e.g., lignin and hemicellulose), or are based on food wastes from traceless or UBQ.

The cost of bio-based plastics has been a major barrier to the development and growth of the market [228,229], but the prices are also decreasing since major food companies and well-known brands are launching or integrating bioplastic packaging products into their portfolios, contributing to the expansion of the production capacities, and the efficiency of the supply chains and all processing steps [230]. In addition, regulation and company-set goals of net-zero CO_2_ emission in the near future also drive bio-based alternatives which were not plausible in the past. Nonetheless, the commercialization of novel (bio)polymers is an arduous task with many challenges to overcome. Notable ones were already discussed above, e.g., price, type, and processability. As for all materials, the properties of bioplastics present several advantages and disadvantages. Some bioplastics present a much higher water vapor permeability compared with normal plastics that, in some cases, can be useful for packed food to release excess vapor or steam [124]. Other disadvantages for food packaging applications include thermal instability, brittleness, poor mechanical properties, and difficulties with heat sealing [231]. On the other hand, these materials are sustainable alternatives with properties, such as biodegradability, and biocompatibility, and they are non-toxic and have a lower carbon footprint compared with oil-based plastics [231]. Furthermore, less obvious factors are the compatibility of the polymer’s recyclability with existing polymers, the volumes at which such a polymer can be produced, and the seasonal and regional differences and availabilities of the starting material. Currently, only materials which can demonstrate their success in all of these aspects will be driven towards a commercial scale and, thus, become a real alternative to current petrol-based packaging.

The availability and seasonality of specific renewable resources needed for the above-mentioned polymers is a key bottleneck in the commercialization process. Successfully scaled bio-polymers have guaranteed this by typically relying on a fermentation stage of sugars from biomass, e.g., sugar cane, bagasse, and hemicellulose streams, as these are easily available all year round in different climates. Novel approaches on different types of renewable resources need to ensure similar resilience against seasonal and regional differences.

Several American and European companies are the top players in what concerns the commercialization of these types of packaging materials. The European Bioplastics Association, in cooperation with the nova-Institute, predicts that the global bioplastics production capacities will increase from around 2.11 million tons in 2020 to approximately 2.87 million tons in 2025 [230]. In addition to the above-mentioned regulations and company goals, supply chain, and resource availability crises, as are currently occurring, provide further pressures and incentives to facilitate increased bio-polymer production in the upcoming years.

## 6. Environmental Impact

In recent years, there is a dichotomy between “biodegradable products are all good” and “petrochemical-based products are all bad”. The use of renewable sources (particularly from agricultural origin) and consumption of less energy are now requirements for the production of industrial products. Therefore, there is an increasing interest in bioplastics due to their renewable nature (raw materials from agriculture instead of crude oil) or biodegradable nature providing less landfilling. Plastics impact the environment and ecosystem during their production, during their service life, and after their disposal, producing contaminants and physical hazards. Bioplastics as potential replacements for petroleum-based polymers require less energy in their production steps and have significantly lower carbon emissions [232,233,234,235]. Therefore, replacing fossil-based polymers with renewable and lower carbon footprint bioplastics is seen to promote the transition to a green bioeconomy with less environmental impact.

For instance, PLA, as a biodegradable polymer, consumes two-thirds less energy in the production step when compared to conventional ones [236], provides no net increase in CO_2_ gas during the biodegradation step [237,238], emits fewer greenhouse gasses when degrading in landfills [239], and reduces greenhouse gas emissions by 25% [240]. Thus, PLA can be considered one of the most suitable candidates for substituting conventional plastics. On the other hand, after composting a biodegradable polymer, the compost can be used as fertilizer or soil conditioner; however, the produced compost can also be a pollution source for soil, water, and groundwater [234]. At the end of their service life, used or wasted polymers are recycled with some losses due to degradation, are incinerated to produce energy with potential environmental pollution, are littered, resulting in environmental hazards, or are landfilled, resulting in carbon or methane emissions over time due to their uncontrolled degradation [70]. Even though, at this disposal cycle, biodegradable polymers are less harmful to the environment compared to petroleum-based polymers, biodegradable polymers are not generally suitable to be landfilled or digested anaerobically due to the potential methane production under anaerobic conditions [200]. The integration of bioplastics with disposal infrastructures includes various facilities, such as composting, anaerobic digestion, recycling, and waste to energy production, as well as their landfilling and debris to the environment. Bioplastics may be alternative materials to petroleum-based polymers; however, clear assessments of the environmental impacts of both petroleum-based polymers and their bio-based counterparts should be explained in greater detail.

In this paper, the environmental effects of bioplastics are examined at two different stages, i.e., “during the production” and “at the end of life”, and the main reasons and key findings are highlighted.

### 6.1. “During the Production”

#### 6.1.1. Land Use—Soil Erosion

Even though biomass is renewable, it requires responsible and optimal use for longer-term sustainability to avoid the overuse of water/fertilizers, soil erosion, reduced land availability, and changing biodiversity [234]. Because of the high competition for the use of biomass by several industries, such as energy (electricity, heat), food/feed (sugar-, starch-based), biofuel, and materials/carbon (wood and paper industry) [241], its use for bioplastic production may create a challenge to strike the balance among the industries. The impact of such use of plants for bioplastic production has gained attention because of direct and indirect land-use changes in agricultural areas or rainforests [234]. Further, the possible loss of soil, which is a non-renewable resource with its complex ecosystem, will result in considerable environmental and economic consequences. For example, the use of forests for agricultural purposes and intensive cultivation, and inappropriate land-use change for more bioplastic production, can result in more soil decomposition [242]. Including unavoidable agricultural or forestry wastes as biomass resources will minimize the competition with land-use for food production [161], which means that agricultural areas or plants remain available and accessible for food production and will be invaluable to the intended bioplastic production [243]. Several researchers have compared the energy use, greenhouse gas emissions, and direct/indirect land-use change for bio-PET [244], bio-LDPE, bio-PVC [245,246,247], and bio-HDPE [248] with their related petroleum-based counterparts. Eerhart et al. [244] studied the energy and greenhouse gas balance for polyethylene 2,5-furandicarboxylate (PEF) bioplastic and compared it to its petrochemical counterpart PET. The non-renewable energy use and greenhouse gas emissions for PEF production were reduced by 40–50% (440 and 520 PJ of non-renewable energy savings) and 45–55% (20 to 35 Mt of CO_2_ equivalents), respectively. Similarly, Alvarenga et al. [246,247] concluded that bio-PVC showed better results than fossil-based PVC based on greenhouse gas emissions and energy savings. Liptow and Tillman [245] showed that bio-LDPE production requires more total energy compared to fossil-based LDPE, although the major share is renewable. For their potential impacts on acidification, eutrophication, and photochemical ozone creation, no significant difference between the two materials has been reported. However, with regard to global warming potential and the contribution of land-use change was reported as decisive. Accordingly, Piemonte et al. [248] studied the land-use carbon emission of corn-based bioplastics with their environmental impact while comparing the results with PE. It was found that the replacement of petroleum-based plastics with bioplastics from waste biomass might sustain the advantages of lowering greenhouse gas emissions. Likewise, Tsiropoulos et al. [249] found 140% lower greenhouse gas emissions for bio-PE than PE and approximately 65% energy savings for bio-PE production. The authors concluded that the combination of some of these measures and the use of biomass for the supply of process steam can further contribute to reducing greenhouse gas emissions.

#### 6.1.2. Loss of Biodiversity

The reduction in global wild species populations, the decrease in crop yields and fish catches, and rising risk of extinction of species, especially farmland birds and insects, are some results of biodiversity loss. The growing interest in using bioplastics will increase land and water use due to bioplastic production [161], and the inappropriate use of pesticides/herbicides/fertilizers will increase deforestation. This trend will result in rising biodiversity loss [242]. Although there have been increasing studies comparing the energy consumption and global warming effects of bioplastics with petroleum-based plastics, more efforts are needed to assess the impacts of bioplastics on biodiversity [161].

### 6.2. “At the End of life”

#### 6.2.1. Recycling of Bioplastics

Reusing the bioplastics, such as polyglycolide, PLA, PHA, bio-PE, and bio-PET, is recommended as a pre-step towards the recycling route, and mechanical recycling should be the following step for as long as possible, until they become low-grade [250]. For instance, bio-PET and bio-PE maintain their good mechanical properties for a decent number of recycles. Chemical recycling should be the route chosen once the polymers become low grade, where each bioplastic has an optimum route with the lowest activation energy [251]. For instance, PLA is recycled via alcoholysis, and bio-PET is recycled via glycolysis, as they produce value-added products [251,252], whereas bio-PE requires pyrolysis to be recycled due to its strong solvent resistance [253,254]. However, the environmental benefits of chemical recycling are deeply debated. Current processes for chemical recycling usually encounter the problems of high cost and high energy consumption and require additional steps for isolation and purification from excessive solvents and catalysts, creating environmental consequences [255]. On the other hand, the presence of biodegradable polymers in municipal waste streams and existing plastic recycling systems may cause problems. For instance, it was stated that the presence of natural fibers or starch might complicate recycling [256]. Even though mechanical recycling can be used a few times without losing the original properties of the biodegradable polymers, such as PLA, when recycled, the possible problems in supplying larger quantities of biodegradable polymer waste make it economically unattractive when compared to petroleum-based polymers [257,258]. The environmental impact of bioplastics can also include an economical angle; however, research has so far focused on the cost of bioplastic production instead of overall cost, including the impact of waste management. As a relatively accepted statement in the recycling systems of bio-based, yet non-biodegradable drop-in plastics, such as bio-PP, bio-PE, and bio-PET, such bioplastics are chemically identical to their fossil counterparts, and can be collected, sorted out, and introduced into the existing recycling streams same as their fossil counterparts. No additional processes or investment costs are expected to recycle these drop-in bio-based plastics [259].

#### 6.2.2. Biodegradation of Bioplastics

The biodegradability and/or compostability of some polymers make a positive effect on the environment by generating carbon- and nutrient-rich compost. Methane gas can be produced via the biological waste treatment of biodegradable polymers at anaerobic conditions [260,261], contributing to global warming as a greenhouse gas [262,263,264,265] that is many times more potent than carbon dioxide [266]. In the aerobic biodegradation of bioplastics in soil systems, degradation products come into contact with soil, and affect the soil microbial environment, where the nutrient uptake by plants and soil physicochemical properties undergo a variety of changes [267]. On the other hand, in marine ecosystem, plastic debris may cause physical hazards for wildlife due to ingestion or becoming entangled in this debris or chemical hazards due to the formation of toxic compounds during oxidation [268].

Release of micro- and nano-plastics into the environment during biodegradation

Macro-, micro-, and/or nano-counterparts of polymers are released into the environment after the degradation or incomplete degradation of polymers. In recent years, the ecotoxicity and the possibility for those particles to enter the living organisms in the food chain are being treated with increasing concern [269]. The environmental persistence of biodegradable microplastics should be shorter than that of conventional plastics; however, they may have similar negative impacts on the environment [270] and their harm is more pronounced when their size decreases. The harmful effects of these particles are found on the biodiversity, growth, reproduction, and wellness of marine organisms. Green et al. [271] studied the effect of PLA microplastics on marine habitats/biodiversity and observed that such microplastics changed the bacteria population and their behavior in marine environments. The effects of biodegradable plastics and their micro counterparts after degradation in aquatic ecosystems has been very recently reviewed elsewhere [272]. On the contrary, Chu et al. [273] recently revealed that PLA-based bio-microplastics may not pose a serious risk for the agroecosystems in the short timeframe spanning from days to months. It was also reported that soil could hold more microplastics (>40,000 microplastic particles/kg of soil) compared to marine environment [274]. The potential environmental impact of microplastics coming from biodegradable polymers were assessed by Shruti et al. [275], and the authors concluded that microplastic formation was inevitable in biodegradable polymers and that their degradation to microplastics needs more research. Straub et al. [276] compared the uptake and effects of microplastic particles from petroleum-based counterparts and from a biodegradable polymer (PHB) in the freshwater amphipod and reported that there were no significant differences in their ingestion and excretion, but that they differed in biological effects. It is inevitable to note that microplastics from bioplastics can be formed faster than in the case of petroleum-based plastics in non-completed degradation systems [277]. Emadian et al. [268] showed that multiple biodegradation environments were not successful for complete biodegradation and, thus, most of the non-biodegraded material is fragmented into micro- or nano-plastics. 

No standardized and accurate methodology is available to quantify the environmental impact of nano- or micro-plastics due to complications caused by a multitude of soil biotic and abiotic processes, the interaction of particles with various components of soil, strong matrix effects, and challenging extraction methods [278]. Even though there is a lack of analytical methods to determine biodegradable microplastics in water, soil, or compost [279], the presence of PHB bio-microplastics was observed by using microscopy [275]. On the other hand, Fojt et al. [280] studied a simple method for the quantification of PHB and PLA microplastics in soils and concluded that biodegradation of plastics might be incomplete and favor microplastic formation.

#### 6.2.3. Incineration with Energy Recovery

Incineration with energy recovery from bioplastics is widely accepted and considered safe with no danger of releasing dioxins or heavy metals [200]. However, as biodegradability is the inherent property of bioplastics, energy recovery should be the least preferred end-of-life option after recycling and biodegradation. It is known that most renewable materials have low calorific values and consume significantly less energy in the production steps, which are positive for the environment [281,282]. However, the value of bioplastics for energy recovery by incineration has not been properly known due to the lack of calorific value determination of biodegradable polymers and the unknown impact of their moisture content on the process. Renewable resources are used for polymer production, which all have a defined circular end-of-life scenario. It is accepted that CO_2_ produced from the incineration of fully bio-based plastics, aerobic composting, or incineration of CH_4_ from anaerobic composting is a net-zero addition to the carbon cycle since, it is used in the photosynthesis to produce new biomass [164,283]. 

#### 6.2.4. Disposal in Landfill

Even though it is accepted that the bioplastic disposal in landfill sites does not require preprocesses such as separation, cleaning, or pre-treatment [284], landfill disposal is considered as the least desirable approach due to the uncontrolled production of the highly potent greenhouse gas methane in landfilled areas. However, in the waste management systems, it has been proposed that such a ‘landfill gas’ can be captured as an energy source, and can then be used as a carbon source input (along with CO_2_ produced during biodegradation) to biodegradation into CO_2_ after its production during biodegradation [70,200]. The degradation of bioplastics in landfill areas consists of different stages [285] and different compounds are produced depending on the type of bioplastics. For example, sugars are produced during landfilling of TPS, and volatile fatty acids are produced during landfilling of PLA and PHB [286]. However, due to the continuous addition of bioplastics into landfills, the phases of degradation overlap and make the determination of the quantity and rate of biogas production in landfills quite complex [287]. During landfilling of bioplastics similar to petroleum-based plastics, the produced biogas will be the critical point that includes the potential uses of biogas for bioplastic production or as a substitute for natural gas [287]. Even though the use of biogas captured from landfills is still not cost-effective, the implementation of biogas capture and utilization is expected to increase by 50% by 2040 [288]. 

## 7. Consumer Research 

The increased consumer demand for sustainable products is fundamental to reaching the proposed goals of minimizing the environmental impact of plastics.

Compared to the plethora of studies on the technical and scientific aspects of bio-based food packaging, contributions from social science consumer research are scarce. This might be due to the fact that, for consumers, the product itself and its price is in most cases more important than the packaging [289,290]. The packaging is rather seen as an information tool [291]. 

Among studies on how consumers respond to bio-based materials, food packaging-related research with 15 contributions comes first, while contributions on bio-based apparel, toys, furniture, and dinnerware, as well as other packaging (non-food) are not as frequent.

In this section, a systematic review on consumer research related to bio-based products based on the PRISMA protocol using Web of Science as our primary database was performed. Our literature search included forward and backward searches, and we added additional articles. Finally, this process yielded 36 studies in total, of which 15 covered food packaging.

Six studies (40%) looked at water bottles, three looked at Coca Cola or other cola products, and two looked at fruit, while other types of food were only represented by one study each (Table 6).

To start on a descriptive level, many authors did not explicitly state on which theory they based their study. Theories that were mentioned were the attitude network approach [293] and the cue utilization theory [304]. Except for two studies that used a mixed methods design [296,297], all other studies were quantitative studies and most of them relied on online surveys.

In line with a large part of consumer research in other areas, the studies under review in this paper often used a quantitative design aimed at explaining stated behavioral intentions, such as willingness-to-pay or intention to purchase by looking at factors that explain these intentions. The factors that were tested can be divided into two broad categories. First, factors pertaining to packaging and its attributes, such as material, recyclability, or labels were considered. Second, factors pertaining to consumers, such as attitudes, norms, and other psychographic or socio-demographic variables were considered.

The dependent variable that studies in our sample sought to explain was primarily willingness to pay (WTP) [292,293,295,298,301,305]. Furthermore, utility [301,302] and preferences [292,294] were closely related to WTP, as well as purchase intention [307]. Other dependent variables were perceived environmental friendliness or, more generally speaking, perceived sustainability [296,297]. One study also examined factors determining correct disposal of biodegradable packaging [297].

The WTP resulting in a surcharge for products packaged in bio-based materials is important information for companies seeking to use these materials in their packaging solutions. Likewise, it was a frequent object of research in our sample. Table 7 summarizes the price premium consumers were willing to pay for bio-based packaging compared to fossil-based packaging. Overall, the range of premiums is very wide, ranging from 8% to 30%. Most of the WTP studies were carried out for water bottles. Overall, it seems that 20% seems to be a premium that is at least a rough approximation for this product category.

One study also asked consumers how they thought about a local ban on expanded polystyrene (EPS) food containers, i.e., not a consumer choice but a regulatory measure [305].

### Influencing Factors

All studies found that consumers harbor more positive attitudes towards bio-based plastic packaging than towards conventional plastics.

The most frequently tested attributes of bio-based food packaging were biodegradability, within six studies [292,293,297,301,302,303], and recyclability within four studies [297,301,302,303], both being seen positively by consumers. Biodegradability also scored positively in other studies not looking at WTP [306]. Furthermore, end-of-life related criteria were more important for consumers than production or transport [277]. Testa et al. [303] tested if third-party certification has an influence but found it to have no significant effect.

The influence of the material for producing bio-based packaging was tested as an influencing factor for WTP in several studies which will be discussed below. Barnes et al. [305], in their study of containers for takeout food, found different preferences in their latent classes, as some preferred sugar-cane, others paper, while corn was not popular among any of the latent classes. Moreover, the material was only the most important attribute for one group. De Marchi et al. [292] tested bio-PET and PLA, with PLA being clearly favored by consumers. Reinders et al. [300] showed that a 100% bio-based product scores much better with consumers than a product with a lower bio-based content.

Local production was tested in one study and, not surprisingly, found to have a positive influence on WTP. Other, less often tested attributes include microwaveability and water resistance [305].

Turning towards consumer attributes as influencing factors, two studies looked at socio-demographics [295,302]. Most other studies that considered consumer attributes examined the influence of various psychographic variables, such as attitudes about bio-based plastics [293,298], environmental attitudes [295], norms [300], trust [295], or knowledge [293,299].

Within their paper, Zwicker et al. [298] did not find attitudes towards bio-based plastics to predict WTP in studies 2 and 4 of their research. However, the attitude towards conventional plastic did, which hints at feelings of guilt. In study 3, both were found to influence WTP but with a very low explanatory power. Guilt was also found to be a driver of WTP [298].

Several studies tested the influence of interventions on choice behaviour, such as nudging and pro-environmental guidance [294], giving information on the environmental effects of different plastics [292,293,295], stimulating feelings of guilt [298], as well as stimulating norms or providing nature pictures or reflective questions [301]. All of these interventions positively influenced the participants’ choice of bio-based packaging.

Finally, the differences between countries revealed in the few cross-country studies [300] make the importance of a differentiated internationalization strategy clear.

The studies under review identified the following barriers to an environmentally beneficial expansion of bio-based food packaging:

A lack of knowledge was frequently discussed to be a barrier. Even with labels clearly indicating a bio-based packaging’s characteristics, consumers seem to have great difficulties in identifying these. In a study by Taufik et al. [297], participants were not able to tell apart bio-based recyclable water bottles and recyclable fossil-based bottles. The participants in the study by Zwicker et al. [298] believed that bio-based plastics are always biodegradable. This false belief can drive acceptance but can also backfire once consumers learn that they have been mistaken. Lynch et al. [299] and Testa et al. [303] pointed out the low level of familiarity with bio-based products in the Netherlands and Italy, while Dilkes-Hoffman et al. [306] and Boesen et al. [296], as well as Zwicker et al. [298] confirm the low level of Australian consumers’ knowledge.

Consumers’ perceptions of the origin of the biomass used to produce bio-based plastics is another potential barrier to further expansion. Zwicker et al. [298] showed that the majority of participants were neutral about whether bio-based plastics contribute to deforestation and food competition. However, nearly 20% (6 and 7 on a 7-point-scale) believed that these materials compete with those used in food production.

Environmental benefits include the correct disposal of the packaging. However, in a study by Taufik et al. [297], 63% of the participants disposed of the compostable bio-based bottle incorrectly. Participants with a higher bio-based product familiarity were more likely to dispose of the compostable bottle correctly. Apparently, the main reason was that participants could not think of plastic and compostable material together. Bio-based plastic was still plastic for them, with all the characteristics they attribute to this kind of material. Similarly, in the study by Dilkes-Hoffman et al. [306], 62% of the participants would dispose of biodegradable food packaging in a recycling bin rather than by composting it. Zwicker et al. [298] (studies 2 and 3 within the paper) showed that consumers find it more important to recycle fossil-based plastic bottles than bio-based bottles. They also showed that consumers in study 3 frequently believed bio-based plastics to be biodegradable, quite the opposite of the findings in the paper of Taufik et al. [297]. Further, in the study by Lynch et al. [299], focus group participants raised the issue of consumers possibly not knowing how to correctly dispose of a bio-based plastic bottle.

What can companies take away from extant consumer research? First, the studies under review have shown that biodegradability and recyclability are important product attributes for consumers. This can be directly applied in companies’ choice of materials and product design, i.e., product strategy. Biodegradability is especially high on the consumer agenda, confirming findings from studies on bio-based packaging in general which have shown that consumers focus strongly on the end of packaging life, i.e., the disposal stage [291]. Furthermore, 100% bio-based products seem to be preferable compared with partially bio-based products. Second, analyses of influencing factors for WTP and differentiated treatments in experiments suggest promising approaches to communication strategy, namely that guilt (when using conventional plastics) seems to be a strong driver of WTP for bio-based products, and that companies can appeal to this emotion in their communication. Along the same lines, norms were shown to be effective; therefore, evoking norms may be a promising element of communication strategy. Moreover, giving pro-environmental guidance in the buying process and pointing out the environmental effects of different types of plastics also have clear effects. However, companies and governments clearly need to educate consumers on how to dispose of bio-based plastics correctly, especially with regard to their biodegradability. Third-party certification did not prove effective; however, since this was tested only in one study, companies should probably consult more studies or include this question into their market research. These hints on communication strategy can not only be applied by companies but also by governments and NGOs in their efforts to persuade consumers to reduce plastics consumption.

Additionally, the pricing strategy can be informed by extant research. The results in Table 7 suggest that a price premium of around 20% could be a good starting point for deliberations on pricing strategies. However, for a final decision, other factors, such as competition and cost, have to be considered.

Looking at the above analysis of consumer research on bio-based food packaging, there are several avenues for further research that seem promising. From a methodological perspective, there is clearly a dearth of qualitative research. Understanding in more detail why consumers prefer certain materials over others and the influences of various attributes, i.e., consumers’ subjective logic, would certainly help to inform both policymakers and marketeers. The study on attitude networks by Zwicker et al. [293] demonstrated how useful this can be. Second, if WTP is to be examined using a quantitative design, it is surprising that direct WTP elicitation methods are still used despite their well-known shortcomings [307]. Choice-based conjoint, which is well-established, and neuroscience-based methods offer interesting alternatives.

However, the consumer–citizen gap must also be considered. While, as citizens, consumers support sustainable packaging, in real shopping situations, the WTP is often much lower, as the citizens then act as consumers, and they have to pay a surplus for more sustainable packaging. This phenomenon has already been studied in depth in the field of animal welfare (cf. e.g., [308,309]).

Concerning potential communication strategies, it would be helpful for companies and governments alike if researchers tested more communication measures, varying both messages and ways of communication, such as text, labeling, or pictures.

## 8. Conclusions 

The interest of researchers has turned in the last two decades to the research of bioplastics, as they are quite promising materials with good properties, such as biodegradability and biocompatibility [310]. The use of biological resources is going to contribute significantly to the production of innovative materials. The advantages of these materials regarding the environmentally friendly solutions are expected to be significant and, to some extent, address the future bioeconomy [83], although mechanical and barrier properties, thermal stability, and water resistance are major problems for many materials, preventing their use in many cases [96]. The application of bioplastics in food packaging compared to conventional materials remains small for reasons related to specific regulations, requirements, price, safety, and their post-use management [86]. This review shows that further research is needed to improve the production of bioplastics and their potential applications, according to different properties, mechanisms of biodegradation, environmental impact, their market and how consumers perceive bioplastics. Governmental economic incentives for these materials and specific rules to limit the use of non-bioplastic materials are mandatory in the future to contribute to the development and commercialization of bioplastics for food packaging and to reduce our dependency on limited petroleum resources. Together with motivated consumers, industry, and also governments, environmental awareness and a willingness to focus on sustainability will definitely contribute to an ecological and circular economy.

## Figures and Tables

**Table 1:** Percentage of imports and exports of plastic waste (adapted from Plastic Atlas [48]; Filiciotto and Rothenberg [49]).

	Malaysia	Thailand	Vietnam	USA	Japan	Germany
**Imports**	11%	6%	5%			
**Exports**				16%	15%	13%

**Table 3:** Studies on bioplastic materials for food packaging developed from fruit by-products during 2017–2021.

Fruit By-Products	Type of Bioplastic Materials	Target Microorganisms	Physical and Mechanical Properties	References
Apricot kernel essential oil	Chitosan films	Reduction in fungal growth on packaged bread slices	Improved water resistance, increased tensile strength	[101]
Grapefruit seed extract	Coating of alginate and chitosan films	Reduced bacteria count by 2 log CFU	Increased barrier properties	[102]
Grapefruit seed extract	Carrageenan films	Large inhibitory zone against *Listeria monocytogenes, Escherichia coli,* and *Bacillus cereus*	Increased water vapor permeability and surface hydrophilicity	[103]
Coconut husk extract	Nanocomposite films or gelatin films	-	Improved water sensitivity	[87]
Mango peel flour and extracts of mango seed kernel	Biodegradable coatings and films	-	Good barrier and antioxidant activity	[104]
Mango kernel extract	Soy protein isolate and fish gelatin films	-	Thicker and more translucent films, increased tensile strength, decreased the water solubility, and increased antioxidant activity	[105]
Apple peel polyphenols	Chitosan films	-	Increased thickness, density, solubility, opacity, and swelling ratio, and antioxidant and antimicrobial activities	[106]
Apple skin extract	Carboxymethylcellulose films	*Listeria monocytogenes, Staphylococcus aureus, Salmonella enterica,* and *Shigella flexneri*	Enhanced mechanical, water barrier, solubility, and antioxidant and antimicrobial activities	[107]
Banana peel extract	Chitosan films	-	Reduced hydrophilicity and excellent antioxidant activity	[108]
Pomegranate peel extract	Chitosan–pullulan composite edible coatings	-	Resistance to water loss and gas transpiration	[109]
Pomegranate peel powder	Gelatin films	*Staphylococcus aureus, Listeria monocytogenes,* and *Escherichia coli*	Increased antioxidant and antimicrobial activities	[110]
Pomegranate peel extract	Zein films	*Staphylococcus aureus, Escherichia coli, Pseudomonas perfringens, Micrococcus luteus, Enterococci faecalis, Proteus vulgaris,* and *Salmonella typhii*	Increased tensile strength and antioxidant Activity, and decreased film solubility and water vapor transmission rate	[111]
Blackcurrant pomace powder	Pectin-based films	*-*	Increased water vapor permeability and antioxidant activity, and decreased tensile strength	[112]

**Table 4:** Studies on bioplastic materials developed from vegetable by-products for food packaging during 2017–2021.

Vegetable By-Products	Type of Bioplastic Materials	Target Microorganisms	Physical and Mechanical Properties	References
Whole potato peel	Active biodegradable films incorporated with bacterial cellulose and curcumin	-	Improved tensile strength, reduced water vapor, permeability, oxygen permeability, and moisture content	[113]
Tomato extract	PVOH films mixed with chitosan and itaconic acid	*Staphylococcus aureus*, *Pseudomonas aeruginosa*, *Salmonella enterica Enteritidis*, and *Salmonella enterica Typhimurium*	Improved physical properties	[114]
Lycopene from tomato extract	Poly-lactic acid films	-	Improved barrier against light and oxygen	[115]
Red cabbage extracts	Gelatin films	-	Increased water solubility, water vapor permeability	[116]
Red cabbage extracts	Active fish gelatin films	-	Improved water and mechanical resistance, and antioxidant activity	[117]
Red cabbage anthocyanins	PVOH and starch, propolis, anthocyanins, and rosemary extract composite films	*Escherichia coli, Staphylococcus aureus*	Improved mechanical strength	[118]
Solid sweet potato by-product	Poly(3-hydroxybutyrate-co-3-hydroxyvalerate) composites	-	Increased thermal stability	[119]
β-carotene from carrot	Films based on cassava starch	-	Increased thickness, and greater stability and solubility	[120]
Tomato-based pigments	PVOH-based biofilms	-	Reduced transparency and increased mechanical resistance	[121]
Okra mucilage	Carboxymethyl cellulose with ZnO nanoparticle nanocomposite films	*Staphylococcus aureus*	Reduced microbial growth, oxidation, and gas production.	[122]

**Table 5:** Overview of existing materials.

Class	Degradability				Barrier		Processability					Feedstock					Application					FC	Price	Prod	References
	M	H	I	N	O	W	I	C	E	T	B	P	N	W	S	C	T	B	P	C	F				
**Proteins**																									
*Zein*	X	X	X	-	B	D	X	X	X	X	X	-	X	X	X	-	?	?	?	X	X	+	B	?	[174,175,176,177,178,179,180,181]
*Gluten*	X	X	X	-	B-C	C-E	X	X	X	X	?	-	X	X	X	-	?	?	?	X	X	+	?	?	[182,183,184,185,186,187]
*Soy*	X	X	X	-	C	D	X	X	X	?	?	-	X	X	X	-	X	?	?	X	X	+	B	?	[188,189,190,191]
*Whey*	X	X	X	-	A-B	A-B	X	X	X	?	?	-	X	X	X	-	?	?	X	X	X	+	C	?	[192,193,194]
*Casein*	X	X	X	-	A	C	?	?	?	?	?	-	X	X	X	-	?	?	?	X	X	+	B-C	?	[195]
*Collagen*	X	X	X	-	-	C	?	?	?	?	?	-	X	X	X	-	?	?	?	X	X	+	C	?	[196]
*Keratin*	X	X	X	-	-	A-B	?	?	?	?	?	-	X	X	X	-	?	?	?	X	X	+	?	?	[197]
**Carbohydrates**																									
*Cellulose-based*	X	X	X		C	D	X	X	X	?	?	-	X	X		X	X	-	X	X	X	+	A-B	B	[198]
*Starch-based*	X	X	X	-	C	C-D	X	X	X	?	?	-	X	?	X	X	X	X	X	X	X	+	B	A	[199,200]
*Chitosan*	X	X	X	-	B-C	C-D	-	X	-	-	-	-	X	X	?	?	?	?	?	X	X	+	B-D	?	[201,202]
*Alginate*	X	X	X	-	B	D-E	-	X	-	-	-	-	X	X	?	?	?	?	?	X	X	+	B	-	[203,204,205]
**Polyesters**																									
*PLA*	-	-	X	-	D	D	X	X	X	X	X	-	X	X	X	-	X	X	X	X	X	+	A-B	A	[168,201,206,207]
*PHA*	X	X	X	-	C	C	X	X	X	X	X	-	X	X	X	X	X	X	?	X	X	+	D	C	[70,208,209,210,211,212]
*PBS*	-	X	X	-	D	B-C	X	X	X	X	?	X	X	-	X	-	X	X	?	X	X	+	B-C	B	[213]
*PBAT*	?	X	X	-	D	C	X	X	X	X	?	X	-	-	-	-	X	X	X	X	X	+	B	A	[214]
*PEF*	-	-	-	X	B	B-C	X	X	X	X	X	-	X	X	X	-	X	X	?	?	?	~	B	D	[164,215,216]
*Bio-PET*	-	-	-	X	D	C	X	X	X	X	X	X	X	X	X	-	X	X	X	X	X	+	A-B	A	[164,217,218]
*PGA*	?	X	X	-	B	B	X	X	?	?	?	X	X	?	?	?	?	?	?	?	?	-	B	?	[219]
**Ethers**																									
*Lignin-based*	?	X	X	-	E	D	?	?	?	?	?	-	X	X	-	-	?	?	?	X	X	~	A-B	?	[220]
**Polyolefins**																									
*Bio-PE*	-	-	-	X	E	B	X	X	X	X	X	X	X	X	X	-	X	X	X	X	X	X	A-B	A	[164,221]
**Lipid-based**																									
*Waxes*	?	X	X	-	E	B-C	X	?	?	?	?	X	X	X	-	-	?	?	?	X	X	X	B	?	[222,223,224]
*Fatty Acid-based*	?	X	X	-	?	?	?	?	?	?	?	-	X	X	-	-	?	?	?	X	X	+	?	?	[225]

General symbols are as follows: no data available (?), applicable (X), not applicable (-); degradability categories are as follows: marine (M), home compostable (H), industrial compostable (I), non-compostable (N); barrier categories are as follows: OTR (O), WVTR (W); barrier values are as follows: A (<1), B (1–10), C (10–100), D (100–1000), E (>1000) [g/m²/d]; processability categories are as follows: injection molding (I), film casting (C), extrusion (E), thermoforming (T), blow extrusion/molding (B); feedstocks are as follows: petrol-based (P), natural biomass (N), monomers from starch/food or feed competition (S), agricultural byproducts/nonfood competition land-use (W), CO_2_/decoupled from land-use (C); application categories are as follows: rigid trays (T), bottles (B), pouches (P), coated cardboard (C), films, wraps, and bags (F); food contact (FC) categories are as follows: approved (+), declined (-), not tested (~); price categories are as follows: A (0.5–2), B (2.1–5), C (6–10), D (>11) [€/kg]; production capacity (Prod) categories are as follows: A (>100), B (51–100), C (10–50), D (<10) [kt/a]; here, PBAT refers to poly(butylene adipate-coterephthalate).

**Table 6:** Overview of packaged food products in the studies under review.

Packaged Products	Number of Studies	Studies
Water	6	[292,293,294,295,296,297,298]
Coca Cola/other colas	3	[296,299,300]
Fruit	2	[301,302]
Juice	1	[303]
Beer	1	[296]
Soup	1	[304]
Takeout food	1	[305]
Food in general (unspecified)	1	[306]

**Table 7:** Overview of price premia in the studies under review.

Studies	Price premium	Method	Remarks
[305]	N/A	Choice-based conjoint	The study only tested bio-based alternatives, no fossil alternatives
[292]	0.07 Euro / bottle (PLA) 0.05 Euro / bottle (bio-PET)	Choice-based conjoint	Percentages were not shown and could not be calculated
[295]	25% PLA over PET (mean) 22–35% depending on treatment 13% PLA over PEF 6–17% depending on the treatment	Direct	Treatments: different messages on the environmental effects of different plastics
[301]	Control group: 23% Other groups: 19–51% (depending on the treatment)	Choice-based conjoint	Treatments: e.g., pictures, normative messages
[293] study 2	21%	Direct	
[293] study 3	18%	Direct	
[298] Study 2	30%	Direct	
[298] Study 3	20%	Direct	
[298] Study 4	8%	Direct	
